# 2660. Evaluating Risk for Bacterial Vaginosis Utilizing an Unsupervised Machine Learning Approach

**DOI:** 10.1093/ofid/ofad500.2271

**Published:** 2023-11-27

**Authors:** Violeta Rodriguez, Yue Pan, Ana Salazar, Nicholas Fonseca, Patricia Raccamarich, Nichole R Klatt, Deborah Jones Weiss, Maria L L Alcaide

**Affiliations:** University of Miami Miller School Of Medicine, Chicago, Illinois; University of Miami Miller School Of Medicine, Chicago, Illinois; University of Miami Miller School Of Medicine, Chicago, Illinois; Division of Infectious Diseases, Department of Medicine, University of Miami Miller School of Medicine, Parkland, Florida; Division of Infectious Diseases, Department of Medicine, University of Miami Miller School of Medicine, Parkland, Florida; University of Minnesota Medical School, Minneapolis, Minnesota; University of Miami Miller School of Medicine, Miami, Florida; Division of Infectious Diseases, Department of Medicine, University of Miami Miller School of Medicine, Parkland, Florida

## Abstract

**Background:**

Clustering methods using machine learning may be useful for identifying variables predicting clinical outcomes. Despite the need to better understand risk behaviors of Bacterial Vaginosis (BV), the most common cause of abnormal vaginal discharge linked to STI and HIV acquisition, machine learning methods have not been used to better understand BV. This study used an unsupervised machine learning algorithm, sidClustering and random forests, to identify clusters of risk behaviors of BV.

**Methods:**

Participants were 402 cisgender women recruited in Miami, Florida, aged 18-45 (median age=31); over half of them were black (56%) and non-Hispanic (43.8%). Participants completed measures of demographics characteristics, sexual and medical history, and intravaginal practices (IVP), and underwent collection of vaginal samples. BV was diagnosed using Amsel or Nugent criteria; abnormal vaginal flora was defined as Nugent score of 4 or above. sidClustering and random forests were used to identify clusters and the most important variables in classifying clusters associated with BV; 135 behavioral variables (including substance use and number of partners) were subjected to analysis.

**Results:**

We identified 4 clusters explained most of the variation in behaviors, and variables were ranked by importance in distinguishing these clusters. Results showed that clusters associated with BV were composed of women who 1) engaged in IVP primarily using water and fingers (Cluster 1: *n* = 108 (26.9%)], 2) engaged in IVP using multiple methods [water, rags, etc.; Cluster 2: *n* = 127 (31.6%)]; 3) engaged in a combination of IVP and other risk behaviors [Cluster 3: *n* = 119 (29.6%)]; and 4) those who did not engage in IVP but engaged in other high-risk behaviors [Cluster 4; *n* = 48 (11.9%)]. Clusters were related to abnormal vaginal flora (*p* < .001). Cluster 2, the cluster with most frequent IVP, had the highest prevalence of BV (64.9%, compared with Cluster 1 (38.0%), Cluster 3 (50.4%), and Cluster 4 (39.6%).

**Conclusion:**

Machine learning methods may be particularly useful in identifying specific clusters of high-risk behaviors, and in developing interventions intended to reduce BV and IVP, and ultimately to reduce the risk of HIV infection among women.

**Disclosures:**

**Maria L L. Alcaide, MD**, Discidium Biosciences: Board Member|Gilead: Honoraria|Merk & Co: Honoraria|Senhwa Biosciences: Honoraria|Virology Education: Honoraria

